# Case report: Treatment of a rare primary cerebellum histiocytic sarcoma with surgery and radiotherapy

**DOI:** 10.3389/fonc.2024.1398350

**Published:** 2024-12-20

**Authors:** Li Yanchu, Zhang Li, Zhang Qiongwen, Duan Jiayu, Wang Feng

**Affiliations:** Head and Neck Oncology Ward, West China Hospital of Sichuan University, Chengdu, China

**Keywords:** histiocytic sarcoma, central nervous system, cerebellum, radiation, surgery

## Abstract

**Background:**

Histiocytic sarcoma originates in various tissues, including the skin, lymph nodes, gastrointestinal tract, lungs, bone marrow, and central nervous system. Primary central nervous system histiocytic sarcoma (PCNSHS) is exceptionally rare, known for its aggressive behavior and poor prognosis. This report describes a case of PCNSHS in the cerebellum treated with surgery and radiotherapy.

**Case presentation:**

A 30-year-old woman presented with progressive dizziness and headache. Magnetic resonance imaging scans showed right cerebellar neoplastic lesions approximately 3.6 cm*3.0 cm with cerebral edema and fourth ventricle and brainstem compression. The patient underwent surgical debulking, and the pathological diagnosis was PCNSHS. Two months after the surgery, the patient underwent adjuvant radiotherapy at a dose of 60 Gy. No tumor progression has been observed during the one-year follow-up period.

**Conclusions:**

This case report provides an example of effective central nervous system control using resection and radiation therapy. A review of the literature confirms that surgery alone or combined concurrent or sequential treatment of radiotherapy and chemotherapy is often used; however, the best treatment plan remains unclear. Moreover, the prognosis is poor, with a median survival of six months. Thus, ongoing research aims to better understand the biology of histiocytic sarcomas and find more effective strategies.

## 1 Introduction

Histiocytic sarcoma (HS) is a rare and aggressive lymphohematopoietic tumor derived from non-Langerhans histiocytic cells of the mononuclear macrophage system (1, 2), which most often involves the lymph nodes and/or gastrointestinal tract (3). Primary central nervous system HS (PCNSHS) is extremely rare, accounting for less than 1% of all lymphohematopoietic neoplasms (4). Although the pathogenesis of HS remains unclear, histologically, PCNSHS is characterized by the infiltration of inflammatory cells in the central nervous system and plays a crucial role in confirming the diagnosis; CD68, CD163, and lysozyme are recognized as typical markers that distinguish PCNSHS from other hematopoietic neoplasms, such as B-cell or T-cell non-Hodgkin’s lymphoma (1, 5–7). Unfortunately, there are no standard or effective treatment strategies for PCNSHS.

Here, we describe a patient with primary cerebellar HS and review the available literature on this aggressive disease.

## 2 Case description

### 2.1 Clinical presentation

**Figure 1:**
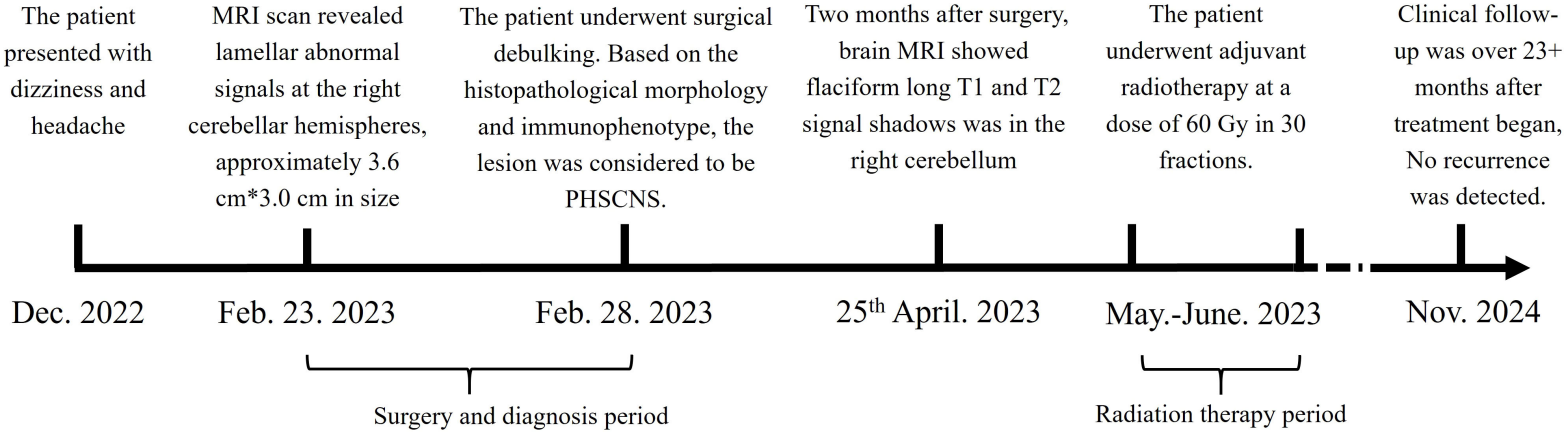
Treatment timeline. The patient suffered from PCNSHS since December. 2022, and diagnosed in February. 2023. After diagnosed, the patient underwent surgical debulking, and received radiation therapy. CR respond reached.

**Figure 2:**
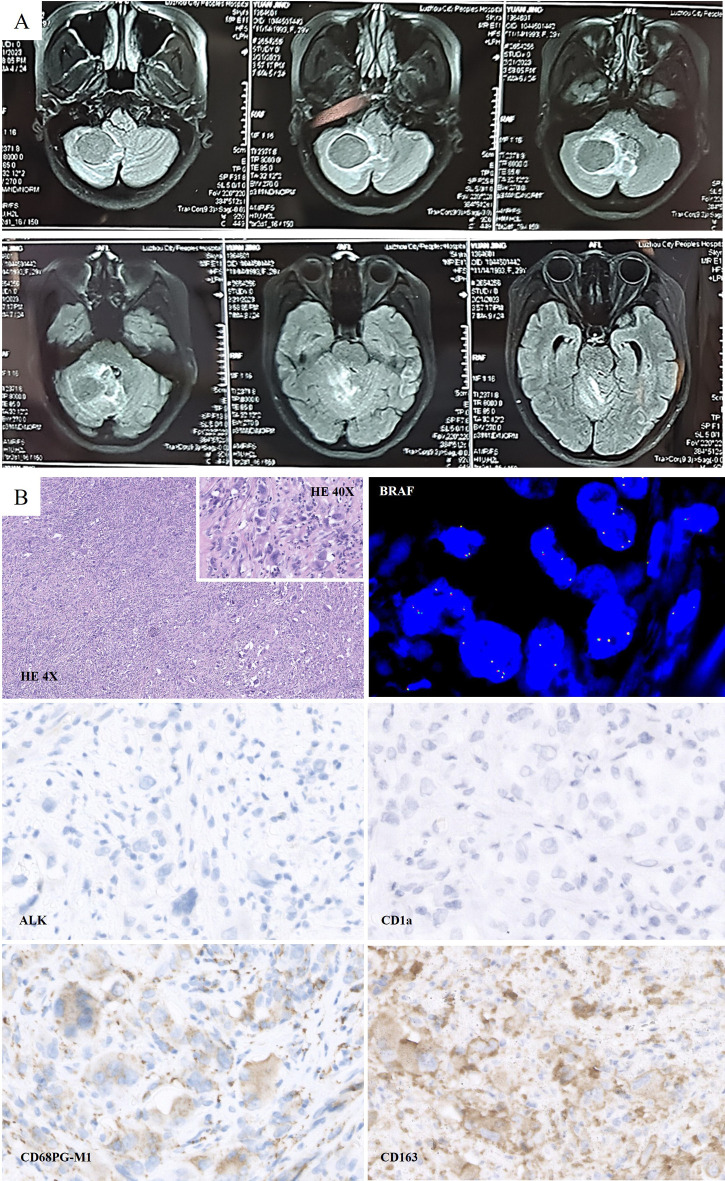
**(A)** Preoperative Magnetic resonance imaging (MRI) scan on February 2023: Lamellar abnormal signals at the right cerebellar hemispheres were detected, which was approximately 3.6 cm*3.0 cm in size. **(B)** Histologically, large cells with abundant cytoplasm were presented. Morphologically, CD163 (+), CD68 (PGM1) (+), CD1a (-), and ALK (-) were detected. FISH test: No *BRAF* gene translocation was detected.

The treatment timeline is shown in **Figure 1**. A 30-year-old woman presented with dizziness and headache that had persisted for three months. On February 23, 2023, magnetic resonance imaging (MRI) revealed lamellar abnormal signals at the right cerebellar hemispheres, approximately 3.6 cm*3.0 cm in size, with clear boundaries, slightly low signal on T1WI, equal or slightly high signal on T2WI, low signal envelope on the edge, and unrestricted diffusion. The lesions showed obvious uniform enhancement on the enhanced scan. Abnormal signals with slightly longer T1 and T2 images were observed around the lesions. The adjacent fourth ventricle and brainstem were compressed and narrowed, respectively. The ventricular system was slightly enlarged, and there were a few symmetrical distributions of slightly longer abnormal T1 and T2 signals around the bilateral ventricles (**Figure 2A**). Preliminary diagnoses were medulloblastoma, pilocytic astrocytoma, or lymphoma.

### 2.1 Treatment and pathology

On February 28, 2023, the patient underwent surgical debulking. Histologically, large cells with abundant cytoplasm were mostly present in cerebellar biopsies. Morphologically, these cells had characteristics of histiocytes, and immunohistochemistry showed GFAP (-), Oligo2 (-), P53 (+), ATRX (+, expressed), HMB45 (-), CK (-), H3K27ME3 (+, expressed), H3K27M (-), CD68 (PGM1) (+), CD163 (+), CD1a (-), langerin (-), and ALK (-). FISH test: No BRAF translocations were detected. Mutations in TERT (P250/P228), BRAF (V600E), or H3F3A/HIS1H3B (K27/G34/K36) were not detected. The examination revealed no clear glial differentiation (**Figure 2B**). Based on the histopathological morphology and immunophenotype, the lesion was considered to be HS.

**Figure 3:**
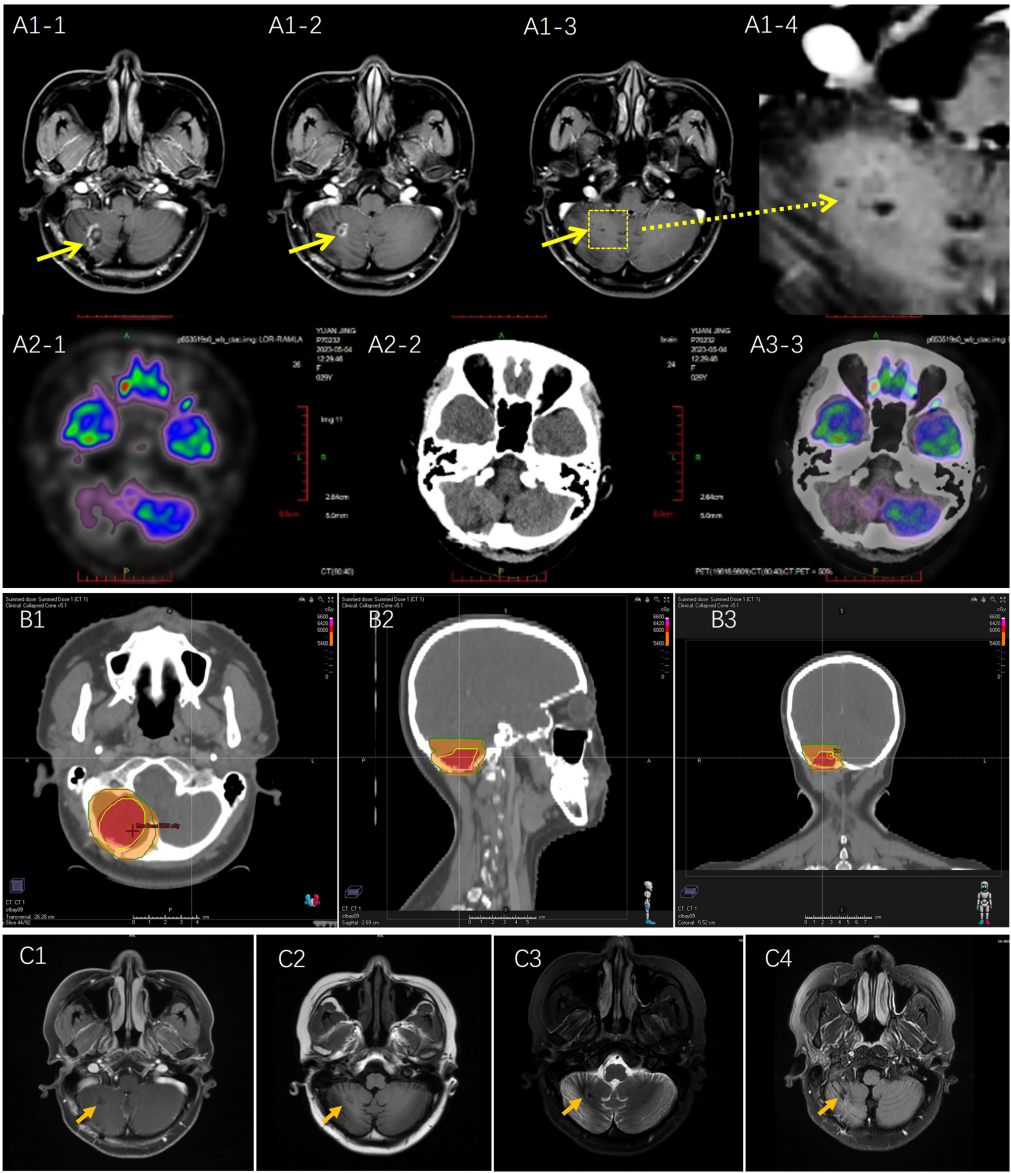
**(A)** Postoperative Magnetic resonance imaging (MRI) and Positron Emission Tomography (PET) scan on April 2023: **(A1)** MRI scan: long T1 and T2signal shadows were observed with annular enhancement; **(A2)** PET scan no metastasis and recurrent tumor were detected. **(B)** Radiotherapy planning: According to RTOG radiotherapy standard, tumor bed as the gross target volume (GTV) was delineated based on MRI T1 enhanced image and T2 Flair image, including postoperative area and edema area, and expanding the GTV range by 2.0 cm became clinical target volume (CTV). Red area: GTV was at a dose of 60 Gy; Orange area: CTV area was at a dose of 54 Gy. **(C)** Brain MRI scan was performed on October 11, 2023.No new recurrent lesion was detected. **(C1)** T1 enhancement; **(C2)** T1 FLAIR; **(C3)** T2 propeller; **(C4)** T2 FLAIR.

On April 25, 2023, two months after surgery, brain MRI showed falciform long T1 and T2 signal shadows was in the right cerebellum, FLAIR represented circular high signal, and annular enhancement was observed on the contrast scan. For staging evaluation, chest and abdominal Computed Tomography (CT), positron emission tomography (PET), and bone marrow examinations were performed (**Figure 3A**). Only the brain lesions were found. To decrease the risk of recurrence, the patient underwent adjuvant radiotherapy at a dose of 60 Gy in 30 fractions. CT with a fixed mask was performed with the patient in the supine position. The RayStation treatment planning system (RaySearch Laboratories, USA) was used for radiation therapy, contouring, and planning. The daily dose of 2.0 Gy was delivered to the patient with an Elekta Linear Accelerator (Edge™) using intensity-modulated radiation therapy plus volumetric modulated arc therapy (**Figure 3B**). The patients were evaluated weekly for radiation toxicity. After adjuvant RT was completed, head MRI was obtained every three months, and no evidence of residual neoplasm or tumor recurrence was found after RT. The patient remains in complete remission (**Figure 3C**).

## 3 Discussion

According to the literature review, PCNSHS is a rare subtype of HS, and its occurrence in the central nervous system distinguishes it from HSs that may occur in other organs. PCNSHS often presents with non-specific symptoms, including headache, seizures, focal neurological deficits, and changes in mental status.

Neurological HS images showed mainly brain parenchymal involvement with single lesions (60%). Commonly, CT is the first choice for HS lesions, which mostly represent high-density lesions due to the high nuclear-cytoplasmic ratio of tumor cells (8); however, MRI is better than CT. According to the MRI scan, the lesions are mostly round or oval, with clear boundaries, and are often located at the junction of the gray and white matter, which may be accompanied by cystic components, necrotic areas (20%), or hemorrhages (4%). This characteristic is helpful for identifying other diseases; however, it still lacks typical characteristics. The lesions mostly appear as T1 equal or slightly high signal, T2 low or slightly higher signal. PET examination revealed hypermetabolic changes (9).

Histologically, PCNSHS is characterized by the infiltration of large pleomorphic histiocytes into the central nervous system (1). Immunohistochemistry plays a crucial role in confirming the diagnosis, with markers, such as CD68, CD163, and lysozyme, being commonly positive, and not expressing specific T or B cell markers, myeloid markers, follicular dendritic cell markers, or epithelial cell markers (1, 8, 10) (**Table 1**).

**Table 1:** Differential pathological diagnosis of histiocytic sarcoma.

	HS	RHP	DCS	LCH	MM
Cytomorphology	Large cells with an epithelioid appearance, featuring kidney-shaped nuclei and copious cytoplasm that is filled with vacuoles	Smooth and uniquely round nuclei characterized by a delicate pattern of chromatin	Spindle to ovoid cells with whorls	Intranuclear clefts, pale nuclei and inconspicuous nucleoli	A monomorphic population of dispersed small round blue cells, with scant cytoplasm
Immunophenotype	CD163(+)	CD163(-)	CD4(+)	Cd1a (+)	S-100(+)
	CD68(+)	CD68(-)	CD68(+/-)	CD68(-)	CD68(-)
	Lysozyme (+)	CK7(-)	CD34 (-)	Langerin/CD207(+)	HMB-45 (+)
	CD1a(-)	CK20(-)	Fascin (+)	CD163(-)	MART-1/Melan A (+)
	CD21(-)		CD21(+)		CD163(-)
	CD35 (-)		CD35(+)		MITF (+)
	CD33(-)		S-100(+)		Tyrosinase (+)

HS, histiocytic sarcoma; RHP, reactive histiocytic proliferations; DCS, dendritic cell sarcomas; LCH, Langerhans cell histiocytosis; MM, malignant melanoma.

Unfortunately, there is no standard therapy for HS or PHSCNS. Patients were treated using multimodal strategies, such as surgery, chemotherapy, and/or radiation therapy. In contrast, surgical resection combined with adjuvant radiotherapy is recommended for patients with single lesions (7). Patients with multiple lesions have a more aggressive clinical course, and combined chemotherapy is recommended; however, the optimal chemotherapy regimen is unclear, and lymphoma treatment regimens are commonly used, such as the CHOP regimen (cyclophosphamide, doxorubicin, vincristine, and prednisone) alone or combined with Etoposide (10). Additionally, genomic sequencing may play an important role in the identification of molecularly targeted agents and immune checkpoint inhibitors. IDBAIH et al. reported a case of neurological HS with BRAF V600E mutation, and the BRAF inhibitor Vemurafenib was used to achieve a good therapeutic effect in the short term (11), and May et al. reported the use of Dasatinib in a case of PHSCNS with platelet-derived growth factor receptor mutation (4). However, even if PHSCNS received treatment, the median survival time is 7.0 ± 0.98 months (95% confidence interval: 5.08–8.92) and an average survival time is 24.07 ± 5.1 months (95% confidence interval: 14.08–34.06) (**Table 2**) (2, 12–15).

**Table 2:** Summary of clinical features of primary CNS HS in recent years.

No.	Year	Age/Sex	Location	Treatment	Follow- up (M)	Outcome
1	1996	20m/M	Leptomeningeal	CT	3	Dead
2	2001	69/F	Parietal lobe	Surgery + RT + CT	8	Dead
3	2001	43/M	Intradural, extramedullary, spinal cord	Surgery + RT + CT	5	Alive
4	2001	11/M	Cerebellum + occipital lobe	Surgery	4	Dead
5	2003	13/M	Occipital- meninges	No treatment	7	Dead
6	2007	53/F	Retroorbital	Surgery + RT	7	Dead
7	2010	71F	Intramedullary spinal	Surgery+ RT	5	Dead
8	2011	52/F	Parietal lobe	No therapy	NA	Dead
9	2012	17m/F	Cerebellar	Surgery + CT	16	Alive
10	2012	55/F	Brain parenchyma	Surgery + RT	4	Dead
11	2012	43F	Parenchyma and spinal cord	CT	10	Dead
12	2012	38/F	Cerebral supratentorial	RT+CT	0.3	Dead
13	2012	62F	Meningeal and cerebellar	Surgery	24	Alive
14	2012	34/M	Meningeal and frontal lobe	Surgery	10	Alive
15	2013	50/M	Occipital and parietal lobe	Surgery+ RT	18	Alive
16	2013	41F	Temporal lobe	Surgery+ RT + CT	42	Alive
17	2013	44/M	Brain lesion (multiple)	CT+RT	6.7	Dead
18	2013	58/M	Brain lesion	Surgery	4.2	Dead
19	2013	16/M	Parietal lobe	Surgery + RT	4	Dead
20	2014	40/M	Temporal lobe	BRAF inhibitor	6	Dead
21	2014	63/F	Trigeminal nerves	CT	0.6	Dead
22	2014	52/M	Frontal lobe	Surgery + CT+ RT	16	Alive
23	2015	15/F	Frontal lobe	Surgery + RT + CT	23	Alive
24	2015	61/M	Sinus	CT+RT	NA	NA
25	2015	23/M	Cerebellopontine	CT+ RT	60	Alive
26	2015	59/M	Brain and spinal cord	CT	2	Dead
27	2016	65/M	Frontal + parietal + spinal cord + meningeal	RT	11	Alive
28	2017	45/F	Leptomeningeal	No therapy	2	Dead
29	2002	46/F	0ccipital lobe	Surgery	NA	NA
30	2015	52/M	Parietal lobe	Surgery + RT+CT	6	Alive
31	2018	55/F	Parietal lobe	Surgery + RT	8	Dead
32	2022	24/F	Right parietal lobe	Apatinib and Anlotinib	10	Dead
33	2022	35/M	Right frontotemporal	Surgery+RT	18	Alive
34	2024	9/F	Anterior falciform region	Surgery	NA	NA
35	Current Case	30/F	Right cerebellar hemispheres	Surgery + RT	23	Alive

CNS, central nervous system; CT, chemotherapy; F, female; HS, histiocytic sarcoma; M, male; RT, radiotherapy.

In this case, the disease was located in the cerebellum, and no metastasis was observed on PET. Thus, only radiation at a dose of 60 Gy was administered after surgery. To date, no recurrence has been reported, and the survival time was 23 months. Meanwhile, such integrated therapies with surgery and radiotherapy might be useful in all cases of sarcoma (16, 17).

## 4 Conclusion

HS is an extremely rare disease with a poor prognosis, and intensive radiation-based chemotherapy is a treatment option. This case demonstrates that patients can benefit from radiotherapy for localized lesions. Furthermore, owing to its rarity, ongoing research aims to better understand the biology of HS and develop more effective treatment strategies.

## Data Availability

The datasets presented in this article are not readily available because of ethical and privacy restrictions. Requests to access the datasets should be directed to the corresponding author/s.
